# The Pain of Unjust Losing. The feeling of injustice and the perception of pain

**DOI:** 10.1192/j.eurpsy.2023.1285

**Published:** 2023-07-19

**Authors:** E. Wojtyna, A. Mucha

**Affiliations:** Institute of Psychology, University of Silesia, Katowice, Poland

## Abstract

**Introduction:**

Social pain is a phenomenon where you feel pain in response to a social stimulus such as feelings of loss, exclusion, and injustice. In today’s world, people often experience unfair treatment. A special case is a situation in which the individual has aroused commitment, but there is no consequence in the form of the expected gratification.

**Objectives:**

The study aims to determine the impact of losing and unjust losing on the perception of pain.

**Methods:**

The study involved 80 people who were randomly assigned to one of the following groups: win, lose, unfairly lose and control. The first three groups participated in a “paper-scissor-stone” game that was created in which they played against a false opponent. The game was constructed in such a way as to obtain the result provided for each group. The “unfairly lose” group received negative points for both a loss and a draw. The control group was only watching the play of two other players. Pre- and post-game pain thresholds and pain tolerance were tested in each group. Pain severity was also assessed. The pain was generated by a thermal stimulus using the TSA-II neuroanalyzer. Pain severity and involvement in the game were analyzed with the VAS scale.

**Results:**

The level of involvement in the game was identical in all three experimental groups. The lowest pain nuisance was observed after the game in the “win” group. The pain was the most strenuous in the group that was unjustly lost. In the group of “unfairly lose”, the pain tolerance threshold decreased after the game.

**Conclusions:**

Feelings of injustice can increase pain and pain sensitivity in people who, after inducing commitment, do not receive fair gratification.

**Disclosure of Interest:**

E. Wojtyna Grant / Research support from: National Science Centre, A. Mucha: None Declared

